# Causes of Industrial Protein A Column Degradation, Explored Using Raman Spectroscopy

**DOI:** 10.1021/acs.analchem.2c03063

**Published:** 2022-11-01

**Authors:** James W. Beattie, Alena Istrate, Annabelle Lu, Cameron Marshall, Ruth C. Rowland-Jones, Monika Farys, Sergei G. Kazarian, Bernadette Byrne

**Affiliations:** † Department of Life Sciences, Imperial College London, LondonSW7 2AZ, United Kingdom; ‡ Department of Chemical Engineering, Imperial College London, LondonSW7 2AZ, United Kingdom; § Biopharm Process Research, Medicine Development & Supply, GSK R&D, Gunnels Wood Road, Stevenage, HertfordshireSG1 2NY, United Kingdom

## Abstract

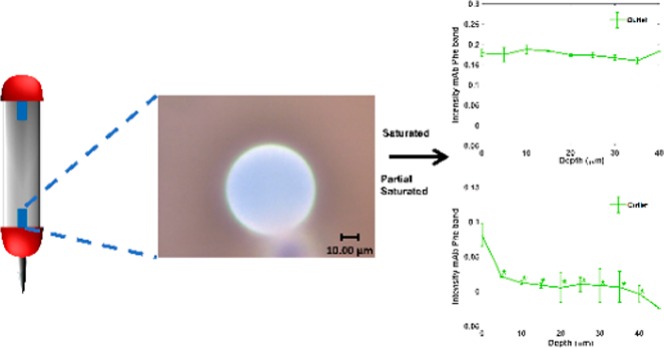

Monoclonal antibodies (mAbs) are used extensively as biotherapeutics for chronic and acute conditions. Production of mAbs is lengthy and expensive, with protein A affinity capture the most costly step, due both to the nature of the resin and its marked reduction in binding capacity with repeated use. Our previous studies using in situ ATR-FTIR spectroscopy indicated that loss in protein A binding capacity is not the result of leaching or degradation of protein A ligand, suggesting fouling is the principal cause. Here we explore binding behavior and resin capacity loss using Raman spectroscopy. Our data reveal a distinct Raman spectral fingerprint for mAb bound to the protein A ligand of MabSelect SuRe. The results show that the drop in static binding capacity (SBC) previously observed for used protein A resin is discernible by Raman spectroscopy in combination with partial least-squares regression. The SBC is lowest (35.76 mg mL^–1^) for used inlet resin compared to used outlet (40.17 mg mL^–1^) and unused resin samples (70.35 mg mL^–1^). Depth profiling by Raman spectroscopy indicates that at below saturating concentrations (∼18 mg mL^–1^), binding of mAb is not homogeneous through used resin beads with protein binding preferentially to the outer regions of the bead, in contrast to fully homogeneous distribution through unused control MabSelect SuRe resin beads. Analysis of the Raman spectra indicates that one foulant is irreversibly bound mAb. The presence of irreversibly bound mAb and host cell proteins was confirmed by mass spectrometric analysis of used resin beads.

## Introduction

In recent years, monoclonal antibodies (mAbs) have become the fastest growing class of biotherapeutics in the U.S. and EU, with 61 first approvals coming on the market between 2014 and 2020 compared to only 34 first approvals between 1997 and 2013.^1^ MAbs are extremely effective due to their high specificity^2^ and low uptake across the blood–brain barrier, resulting in limited off-target effects.^3,4^ Therapeutic mAbs are used to treat a range of chronic and acute conditions^1^ including triple-negative breast cancer,^5^ Ebola,^6^ COVID-19,^7^ and multiple sclerosis.^8^ Immunoglobulin type gamma (IgG) is the dominant subclass of commercially available therapeutic mAbs. Adalimumab, used to treat rheumatoid arthritis, was the bestselling drug of 2018, generating sales worth $19.9 billion.^9^ Although the global market in therapeutic mAbs is experiencing record sales, their very high cost (on average $100,000 per patient per year) limits patient access to these life-changing and life-saving drugs.^10^ Approximately 80% of the cost of therapeutic mAbs is attributable to downstream processing,^11^ essential to ensure the final product meets strict regulatory purity requirements.^12,13^

Typically mAbs are produced recombinantly in Chinese hamster ovary (CHO) cells^2^ and secreted into the growth media. The resultant cell culture fluid contains high levels of mAb as well as host cell proteins,^14^ media components, cellular DNA, and viruses^12,13^ which can cause highly undesirable immune responses in patients.^15^ Effective purification of mAbs from cell culture fluid involves a number of different steps, with the key step exploiting protein A affinity chromatography to remove the vast bulk (98%) of contaminants.^16^

Protein A affinity chromatography utilizes*Staphylococcus* protein A (SPA) as a ligand to capture mAbs with high specificity. The reversible interaction between SPA binding domains and the constant heavy domains 2 and 3 (CH2-CH3) of the Fc region of IgG involves a combination of hydrophobic interactions, salt bridges, and hydrogen bonding.^17^ The mAb is bound to SPA immobilized onto chromatographic beads at neutral pH. Reduction of the pH to ∼3 results in protonation of histidine 137 of protein A and histidine 435 of IgG and subsequent release of the bound mAb due to electrostatic repulsion.^18^ To remove strongly bound contaminants in the column after repeated use, a cleaning in place (CIP) step is used, typically employing up to 0.5 M NaOH.^19−21^

Protein A affinity chromatographic resin costs over double that of other resins utilized in downstream processing and is thus responsible for most of the downstream processing costs.^11,22^ The high costs are exacerbated by lifetime degradation of protein A resins, discernible as a loss in mAb binding capacity over time.^11,23^

A range of analytical techniques have been used to better understand the cause of lifetime degradation of protein A resin including confocal laser scanning microscopy (CLSM),^24,25^ scanning electron microscopy (SEM),^24^ mass spectrometry,^23^ and, by our groups, attenuated total reflection Fourier transform infrared (ATR-FTIR) spectroscopy.^20,26,27^ CLSM showed an increase in both accumulation of foulant and bead pore blockages in protein A resin beads as a function of increased usage, a finding supported by SEM.^25^ CLSM, although a powerful technique, requires fluorescent labeling which can change the absorption behavior of proteins.^28^ As a result, fluorescently labeled proteins can be displaced by nonfluorescent proteins with stronger binding, affecting interpretation of CLSM results. An increase in irreversibly bound host cell protein (HCP) and mAb contaminant with an associated decrease in protein A resin binding capacity detected via liquid chromatography–mass spectrometry (LC-MS/MS) has also been reported. The analysis, carried out following an increasing number of purification cycles, revealed marked HCP accumulation after 80 bind/elution steps.^23^ ATR-FTIR spectroscopy has been used to assess SPA ligand stability after repeated use^27^ and the effects of extended CIP exposure on protein A ligand^20^ as well as for quantifying mAb bound to the surface layer of resin beads in column and offline.^26,27^ ATR-FTIR spectroscopy is nondestructive and label-free, providing a chemical footprint which contains information on the structure of a protein sample. However, ATR-FTIR spectroscopy is limited to probing only the surface layer (∼5 μm) of resin beads,^29^ which can be up to 120 μm in diameter.

Confocal Raman spectroscopy represents a powerful alternative, as it is able to probe much deeper into samples,^30^ while, like ATR-FTIR spectroscopy, providing detailed chemical information and giving insights into protein secondary structure.^31,32^ Additional features are also detectable in Raman spectra, such as amino acid residue side chains, and particularly aromatic amino acid residue side chains.^33^ The ability to view additional features allows for more bands to be utilized in distinguishing between the desired protein analyte peaks and those obtained from protein A ligand and the matrix. Raman spectroscopy is also a label-free technique, meaning native forms of the protein can be studied.^34^ This technique has been used to explore the distribution of ligands bound at both the surface and within pore regions of agarose beads.^35^ Previous studies have applied confocal Raman spectroscopy to the study of mAb binding to a cation resin, Fractogel EMD SO_3_, at different ionic strengths.^36^ The study demonstrated that it was possible to use this approach to obtain a depth profile for mAb binding to the resin beads and show that mAb binding occurred preferentially at higher ionic strength conditions. A subsequent study using the same approach revealed that mAb desorption from the Fractogel EMD SO_3_ occurred at pH 5.0, while at pH 4.0, the mAb adopted a conformation that stayed bound to the resin.^37^ Here, we describe the application of confocal Raman to depth profiling of mAb binding to used and unused MabSelect SuRe, a protein A resin commonly used for biotherapeutic antibody purification. Our results show that mAbs irreversibly foul MabSelect SuRe resin reducing the pore size of the beads and thus disrupt pore diffusion, the primary driving force of adsorption. Partial least-squares (PLS) regression in combination with Raman spectroscopy shows that the spatial location of resin within a column influences the extent of reduction in binding capacity. The results show that the binding capacity decreases to a greater extent in inlet resin samples and this is reflected in more marked changes in adsorption profiles compared to outlet and unused resin samples. The combination of Raman spectroscopy and mass spectrometry used here provides a unique insight into the identity of irreversibly bound contaminants and how the presence of these molecules on the resin not only affects overall static binding capacity within a column but also binding of the mAb within individual resin beads. These findings have the potential to inform new ways of working in terms of large-scale therapeutic antibody production. Our study also highlights the power of Raman spectroscopy as a technique for probing purification events at depth in protein A and other chromatographic resin beads.

## Experimental Section

### Static Binding Capacity Measurements of Protein A Beads

IgG4 (cB72.3) was prepared, isolated, and concentrated to 10 mg mL^–1^ as described previously.^20,26,27^ MabSelect SuRe resin (Cytiva, UK) was analyzed in this study. Used MabSelect SuRe samples, harvested from the inlet and the outlet of an AXIChrom 981 mL column after 25 cycles of purification, were donated by GSK Biopharm Process Research as described previously.^27^ A MediaScout ResiQuot (ATOLL, Weingarten, Germany) was used to pack and equilibrate 20.8 μL (*V*_resin_) of resin into the wells of a 96-well Supor filter plate with a pore size of 0.45 μm. Purified IgG4 was diluted with phosphate buffer (pH 7.4, 50 mM PBS, 150 mM NaCl) to prepare a range of concentrations (*C*_O_) of 1, 2, 3, 4, 5, 6, 7, and 9 mg mL^–1^. Aliquots (200 μL, *V*_sample_) of IgG4 solution at each concentration were individually added to packed resin samples in filter plates which were then mixed for 45 min at 1000 rpm at ambient temperature. The filter plate was centrifuged at 493 *g* for 2 min to remove the excess unbound IgG4 (*C*_eq_) into individual wells of a 96-well plate. The amount of excess unbound IgG4 which flowed through at each loaded concentration was determined on a nanodrop lite at OD_280_ nm with *E*^1%^ = 13.7. The concentration of unbound mAb in the flow through was used to determine mAb binding capacity (*Q*) of the resin using the mass transfer equation^38^ given below (eq 1).
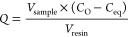
1

### Confocal Raman Spectroscopic Analysis of Protein A Resin Samples

Individual protein A resin samples (∼10.4 μL), both used and unused, from the SBC experiments were carefully applied to a glass slide using a polypropylene spreader. The beads were visually inspected at 50× magnification (Figure S1) to ensure the beads were not damaged during this step. The glass slide containing the sample was placed onto the sample stage of a SENTERRA II (Bruker, Germany) Raman spectrometer in reflectance mode. Individual protein A beads were measured using a laser at 532 nm with a power 12.5 mW and 50× objective. The spectral resolution was 4 cm^–1^. Samples were measured with 24 coadded scans and an integration time of 3 s. A schematic of the experimental setup can be seen in Figure S2. The generated Raman spectra were imported into the software package Orange^39^ equipped with the Quasar addon.^40^ As an additional control, we also carried out a single point measurement on nonfunctionalized agarose beards (Pierce) to generate a reference spectrum.

For confocal Raman measurements of depth profiling of beads, protein A resin samples with no mAbs added were measured in 10 μm steps along the *z*-axis. The same experiments were carried out for resin samples with mAbs added (saturated and partially saturated) with the exception that measurements were taken in 5 μm steps along the *z*-axis. Raman spectra were rubber band baseline corrected between 600 and 1800 cm^–1^ followed by integration between 600 and 1800 cm^–1^. The *z* spatial location with respect to the bead surface was determined by observing the maximal intensity when plotting integration as a function of stage position (Figure S3). The spectra were then vector normalized to compare protein content between measured scattering volumes and different depths of the bead.

For quantification, two individual protein A beads from each of the resin samples generated for SBC analysis were measured. Identification of the spectra with maximal intensity and thus corresponding to the bead surface layer was determined by two co-added scans and the video-assisted measurement mode in OPUS. Raman spectra were rubber band baseline-corrected between 600 and 1800 cm^–1^ and vector normalized. Spectra were imported into MATLAB (MathWorks, Natick, USA) for partial least-squares (PLS) regression with the plsregress function which uses the SIMPLS algorithm.^41^ Where appropriate 1-way ANOVA was carried out on the spectra.

### Mass Spectrometry Analysis of Foulants

The unused MabSelect SuRe and the GSK used inlet and outlet (100 μL, 50% v/v) resin samples were vortexed at 2500 rpm for 10 min. A solution (50 μL) of urea (8 M) and ammonium carbonate (100 mM) was added to the resin slurry followed by addition of dithiothreitol (2 μL, 450 mM) and the sample mixed at 1000 rpm at room temp for 1 h. Iodoacetamide (20 μL, 100 mM) was then added, and the samples were incubated for 15 min at room temp with mixing at 1000 rpm. Trypsin (128 μL, 39.06 μg/L) was added and the resultant slurry mixed at 1000 rpm, 37 °C for 1 h as described previously.^23^ A nano-LC Orbitrap mass spectrometer was used to analyze the samples. The data obtained using a Protein Metrics BYOS platform. A MS/MS score of 200, MS1 score of 0.95, and FDR of 1% were used to filter out false positives and contaminants. Peptides were identified by searching the UniProt database.

## Results

### Quantification of MAbs Bound to Protein A Affinity Resin Using Confocal Raman Spectroscopy

The SBC protocol described here allows for both controlled loading of mAbs onto resin samples and assessment of the overall performance of used and unused MabSelect SuRe samples. In this study we evaluated mAb binding to MabSelect SuRe resin samples obtained from the inlet and outlet of a pilot scale column from GSK that had undergone 25 purification cycles and compared these samples with a control of unused MabSelect SuRe resin.

**Figure 1:**
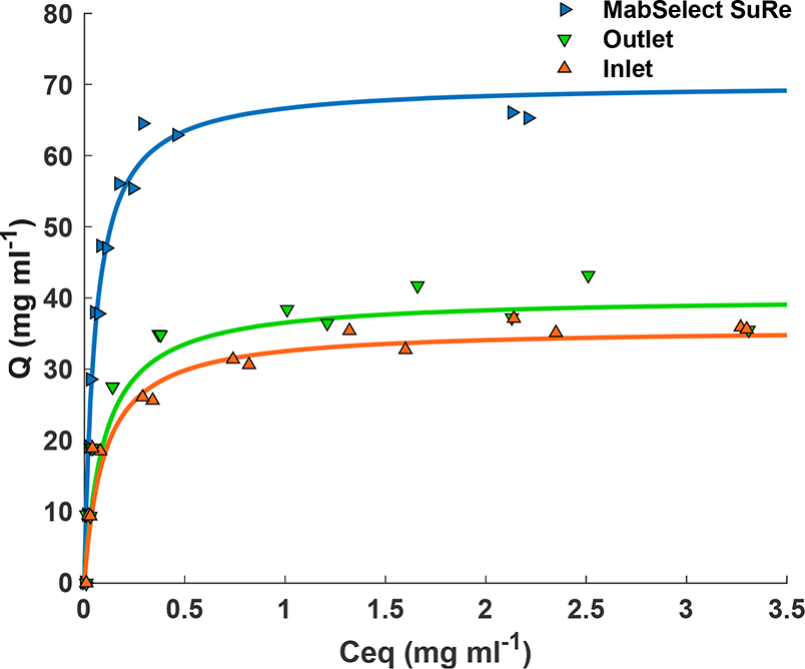
Static binding capacity measurements of resin samples fitted to a Langmuir adsorption isotherm. Unused MabSelect SuRe (blue) and used MabSelect SuRe samples obtained from the inlet (orange) and outlet (green) of an AXIChrom 981 mL pilot scale column which had been subject to 25 purification cycles were analyzed. Each measurement was performed in duplicate.

The unused MabSelect SuRe gave a maximum binding capacity (*Q*_max_) of 70.35 mg mL^–1^. This value is higher than reported elsewhere in the literature (64 mg mL^–1^),^19,38,42^ including in our earlier studies.^20,27^ We are not sure why binding capacity is so high in this case, but it might be a function of different batches of resin. In contrast, used samples from the inlet and outlet of a used pilot scale column exhibit markedly reduced maximum binding capacity values of 35.76 and 40.17 mg mL^–1^, respectively (Figure 1), consistent with values we reported previously.^27^ The dissociation constant (*K*_d_) of mAb for the protein A resin samples was 0.1 mg mL^–1^ for all samples.

The individual samples prepared for SBC analysis were subsequently used for confocal Raman spectroscopic measurements. The amide I band at 1655 cm^–1^ in the confocal Raman spectra is indicative of the primarily α-helical structure of protein A. This is similar in both the unused and used resin samples (Figure S4). This agrees with our earlier ATR-FTIR spectroscopic analysis, which revealed that changes in secondary structure of protein A are not responsible for the drop in SBC observed for the used resin samples.^27^

**Figure 2:**
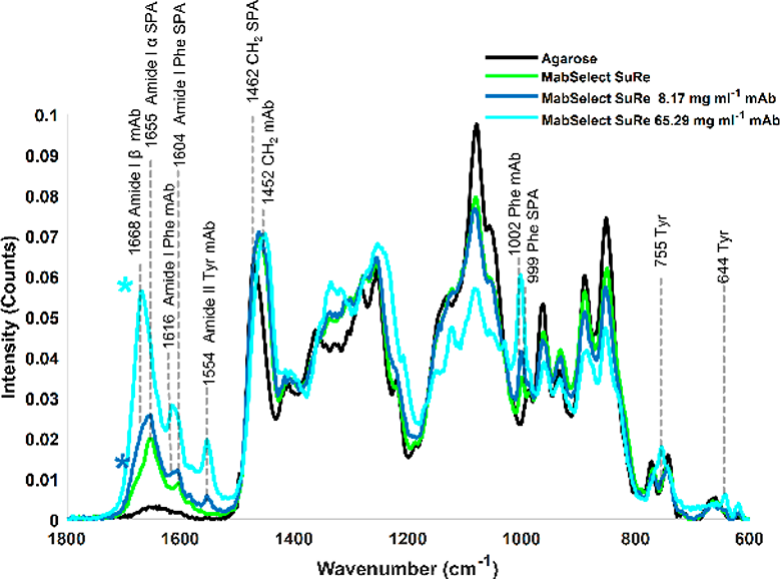
Raman spectra of Pierce nonfunctionalized agarose resin (black), MabSelect SuRe with no mAbs loaded (green), MabSelect SuRe with a nonsaturating concentration (8.17 mg mL^–1^) of mAbs loaded (dark blue), and MabSelect SuRe with a saturating concentration (65.29 mg mL^–1^) of mAbs loaded (light blue). Assigned spectral bands of protein A and bound mAb are labeled. * = a significant difference in spectral intensity, *p* < 0.05, relative to the control of MabSelect SuRe. Example spectra are representative of *n* = 2 beads from each individual resin sample.

In order to accurately perform band assignment of the measured spectra, a combination of literature values^32,36^ and spectral comparison was used (Figure 2). MabSelect SuRe resin with/without mAbs bound and the nonfunctionalized Pierce agarose were used to identify and differentiate between protein bands in the measured spectra.

SPA has a distinct secondary structure detectable as the amide I band at 1655 cm^–1^ (Figure 2, green spectrum), not present in the nonfunctionalized agarose (Figure 2, black spectrum). The change in conformation of SPA upon mAb binding is detectable in the Raman spectra as a shift in the amide I band from 1655 to 1668 cm^–1^ (Figure 2 light and dark blue spectra). Further structural differences in protein A upon mAb binding are apparent from the shift in the phenylalanine (Phe) bands from 999 and 1604 cm^–1^ to 1002 and 1616 cm^–1^, respectively (Figure 2, light and dark blue spectra). Tyrosine bands at 1554, 755, and 644 cm^–1^ are also characteristic spectral features of mAb binding to protein A (Figure 2, light and dark blue spectra). The Phe and Tyr bands become both more prominent and more defined with increasing mAb concentration (Figure 2, dark blue spectrum). There is a significant increase in overall spectral intensity upon addition of both subsaturating and saturating concentrations of mAb (Figure 2, light and dark blue spectra, respectively).

PLS regression was used to quantify the bound mAb and to identify those spectral bands that correlate to a change in the binding capacity of resin (*Q*, the *y* variable for the PLS analysis). Our analysis utilized the confocal Raman secondary structural features, such as the amide I band, as well as tertiary structural bands representing amino acid side chains, such as Phe, in order to quantify mAbs bound as shown in our PLS loading plots (Figure S5). Raman spectra of unused MabSelect SuRe resin samples with known concentrations of bound mAb were used as the training data set for PLS regression. After leave one out cross-validation (LOOCV) was applied, analysis of root-mean-square error cross-validation (RMSECV), R2, Q2 and loading plots (Figure S5) was used to pick five latent variables for the model. The first latent variable of the model which accounted for 82% variance, utilized multiple peaks in the Raman spectrum, most notably both amide I peaks at 1668 and 1616 cm^–1^ as well as the Phe band at 1002 cm^–1^. The next 4 latent variables accounted for ∼4% variance (Figure S5) each. Five latent variables resulted in a total variance coverage of 98% (R2) with a decrease in coverage after cross-validation to 89% (Q2) (Table 1). To compare with the SBC approach of quantification, the coefficient of variation (%CV) for the model against the training data was found to be 9%, and %CV changed to 20% once LOOCV was applied (eq 2 as shown below).
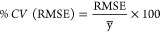
2y̅ is the actual average measurement.

**Table 1:** Statistics of the Five Component PLS Model^a^

Data set	Statistic	Value
Training	R2	0.98
	RMSEC	3.19 mg mL^–1^
LOOCV	Q2	0.89
	RMSECV	7.36 mg mL^–1^
Test	Q2	0.90
	RMSEP	7.18 mg mL^–1^

a Observed (SBC *Q* value) vs fitted (PLS prediction) mAb concentrations were used to assess the model.

The model was applied to the test data set consisting of 64 spectra in total; 32 spectra each for the GSK used inlet and outlet samples. Each of the 32 measurements included 2 individual beads from each SBC condition (8 mAb concentrations for each of *n* = 2 SBC experiments). Test resin samples were loaded with mAbs up to saturation (41.73 mg mL^–1^). Application of the model to the test data set yielded RMSE and variance values of 7.18 mg mL^–1^ and 0.90, respectively, a performance similar to that of LOOCV (Table 1 and Figure S6).

### Depth Profiling of Protein A Affinity Resin Beads

**Figure 3:**
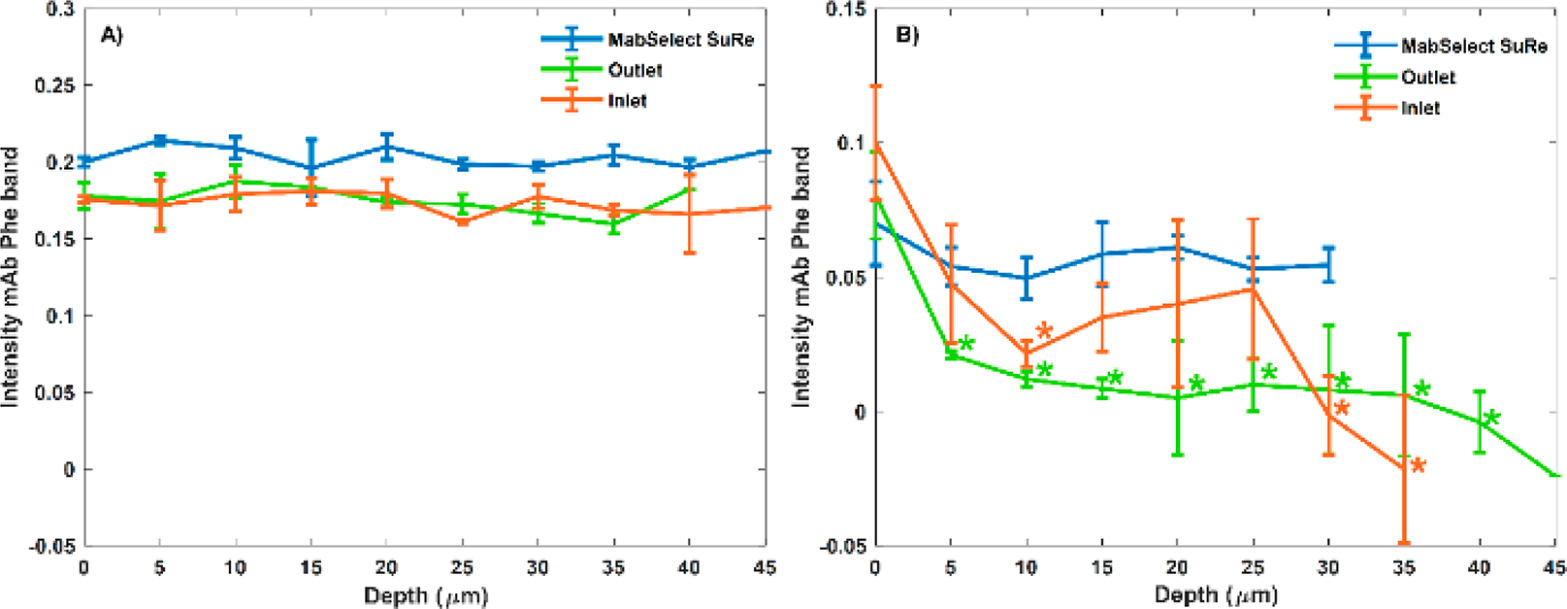
(A) Protein A resin depth profiles of adsorbed mAb at saturating concentrations (mean SBC calculated concentration (*Q*) of resin samples; MabSelect SuRe = 65.68 mg mL^–1^, inlet = 37.57 mg mL^–1^, outlet = 38.80 mg mL^–1^). (B) Protein A resin depth profiles of adsorbed mAb at below saturating concentrations (mean SBC calculated concentration of resin samples: MabSelect SuRe = 19.04 mg mL^–1^, inlet = 18.46 mg mL^–1^, outlet = 18.85 mg mL^–1^). The Phe band at 1002 cm^–1^ was integrated (993–1010 cm^–1^) as a means of observing mAbs bound to the resin samples. Blue = MabSelect SuRe, green = GSK used outlet, orange = GSK used inlet. Data shown are the average of *n* = 2 measurements ± standard deviation (SD). Saturated samples (A) showed no significant difference in intensity at different probed depths. Used resin (inlet/outlet) showed significant differences (*p < 0.05) in mAb binding at different probed depths compared to the surface of bead, when mAb was loaded at below saturating concentrations (B). Spectra as a function of depth for the above depth profile of Saturated MabSelect SuRe, partial Saturated MabSelect SuRe, and outlet samples are shown in Figure S8A–C.

Depth profiling has been used previously to explore mAb adsorption profiles during chromatography polishing steps, normally used after protein A affinity chromatography.^36,37^ Here we depth profiled protein A resin loaded with known concentrations of mAbs. When depth profiling nonuniform sized resin beads it was important to consider that depth resolution decreases as deeper areas of sample are probed as defined by Everall.^30^ With confocal Raman, samples appear to have an apparent depth less than the true depth. For example, if we measured a bead of 50 μm in diameter at 40 μm depth with our setup, we would be illuminating an extra 11.2 μm and thus beyond the bead into the glass slide below. This meant fewer points in the *z* direction could be used, observed in Figure 3B where the MabSelect SuRe and inlet samples cut off.

Depth profiling shows that when unused MabSelect SuRe is loaded with mAbs at or below saturation concentration, binding occurs homogeneously throughout the resin (Figure 3A,B). However, below saturation concentrations of mAb (∼20 mg mL^–1^), the used resin samples give a heterogeneous adsorption profile, with significantly reduced binding activity occurring in the core of the bead (Figure 3B).

Interestingly, once a saturating concentration of mAb is loaded, a fairly homogeneous depth profile is observed in the used resin samples. One way analysis of variance (ANOVA) showed that for each sample the Phe band intensity did not significantly change at each probed depth for each resin sample (Figure 3A). The Phe band is less intense for both the used inlet and outlet samples at both test concentrations, indicating less mAb binding.

### Identification of Protein A Foulants

Based on our previous ATR-FTIR spectroscopic analysis,^27^ indicating that the drop in binding capacity is not due to either leaching or denaturation of the protein A ligand, and other published reports,^23^ we hypothesized that reduction in protein A resin binding capacity is due to fouling of the column. However, the precise nature of these foulants remains unknown.

**Figure 4:**
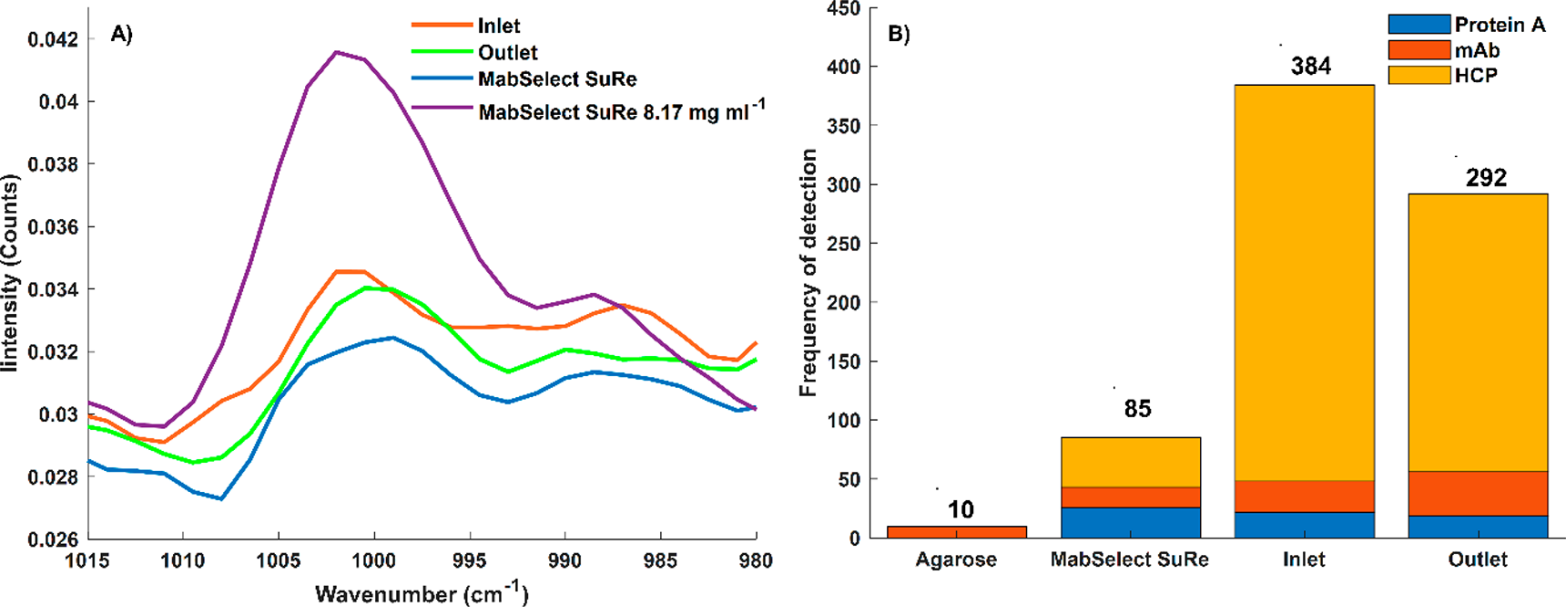
(A) Confocal Raman spectra obtained from different protein A resin with no mAbs loaded. The Phe band region from spectra obtained from MabSelect SuRe (blue), GSK used outlet (green), GSK used inlet (orange) and MabSelect SuRe with 8.17 mg mL^–1^ mAb bound (purple). The Phe band at 1002 cm^–1^ obtained from the Raman spectra was integrated (993–1010 cm^–1^) as a means of detecting mAbs bound to resin samples. Example spectra are representative of *n* = 2 beads from each individual resin sample. (B) LC-MS/MS analysis showing the frequency of detection of peptides in different protein A resin samples with no mAbs loaded. Peptides detected for each sample are grouped into category’s protein A (blue), mAb (red), and HCP (yellow). HCP in this figure refers to all identified peptides that are not protein A or IgG based. Nonspecific mAb peptide fragments are also detected at low frequency in the control samples of agarose and unused MabSelect SuRe and these are likely to be due to contamination during the MS procedure. Spectra as a function of depth for inlet resin beads with no mAbs loaded are shown in Figure S8.

Careful analysis of the Raman spectra in the presence of no mAb indicates a slight but noticeable increase in the Phe band for the used resin samples compared to the unused MabSelect SuRe (Figure 4), indicating the presence of low concentrations of Phe containing proteins within the used resin. Increased Phe band intensity is only detectable at specific depths within the beads. For example, in the case of the inlet resin sample, this increase is only observed at a measurement depth of 30 μm into the beads (up to 38.4 μm considering depth resolution) with a ∼55 μm diameter. This depth is close to the surface of the bead where contamination is more likely, since a greater number of adsorption events will occur on and close to the surface than in the core of the beads (Figure 3B).^36^ A similar result is seen for the outlet resin, where the highest Phe band intensity is observed at 40 μm from the bead surface.

Comparison of the Phe band peak shape for each sample reveals that the inlet Phe band location at 1002 cm^–1^ matches that of MabSelect SuRe + mAbs (Figure 4A), indicating the presence of irreversibly bound mAb to this resin sample. The SPA ligand of MabSelect SuRe has a Phe band which resides at 999 cm^–1^. The less degraded outlet resin has a Phe band location between that of the SPA and mAb Phe bands, likely due to less fouling material being present, with both 999 and 1002 cm^–1^ Phe bands contributing to the resulting band. Further nano-LC-MS/MS analysis confirmed the presence of irreversible bound mAbs as well as a range of other HCPs (Figure 4B and Table S1) in both used resin samples, with the inlet having 92 more peptide detections than the outlet sample. Peroxidases, known HCPs,^43^ were present in high peptide frequency. Generation of a 75 cycle resin sample showed that following this level of use the amounts of HCPs continue to accumulate, but the amount of irreversibly bound mAb stayed more or less the same (Figure S7).

## Discussion

Loss of binding capacity significantly reduces the lifespan of industrial protein A columns,^11,23^ contributing significantly to the overall cost of therapeutic antibody production, and thus continues to limit availability of these life-changing and life-saving drugs. Here we explored the possible causes of the loss of static binding capacity (SBC) of used MabSelect SuRe resins using confocal Raman spectroscopy. The results confirm our earlier findings, that spatial location of resin within a column determines the degree to which the binding capacity degrades with use. Sample from a column inlet exhibits lower binding capacity, than that at the outlet, likely due to a larger number of adsorption events occurring at the inlet.

The Raman PLS model was able to predict a wider range of bound mAb concentrations than the PLS model obtained from our previous ATR-FTIR spectroscopic analysis.^27^ However, the %CV (RMSE) for the Raman model (20%) was higher than the ATR-FTIR spectroscopic model (18%), indicating that the Raman model is slightly less accurate at predicting mAb concentration. Importantly, industry standards (<20%) for predicting binding capacity were met by the ATR-FTIR spectroscopic model with the Raman model on the threshold. The Raman model underpredicted test data when actual bound mAb concentrations were below 20 mg mL^–1^ (Figure S6). Raman spectroscopy has an advantage over ATR-FTIR spectroscopy,^27^ as it is able to probe past the surface of resin beads to explore causes of column degradation. Thus, as demonstrated by the current study, Raman spectroscopy provides additional information that is not possible to obtain with ATR-FTIR spectroscopy.

Previous studies show pore blockages occurring in protein A resin after use. CLSM, for example, revealed that fluorescently labeled mAbs bound to the resin but were not completely removed by typical CIP procedures.^23,44^ Given that CLSM requires fluorescently labeled mAb and the presence of the fluorophore alters the binding properties of the mAb, it was not clear from these earlier studies whether the pore blockages observed were representative of what would happen in a normal column or were occurring as a result of the modified protein. Here we were able to analyze nonlabeled, native protein and confirm that the drop in SBC of the protein A resin is due to buildup of contaminants in the resin pores that both reduces overall binding and limits where mAb binds under nonsaturating concentrations of mAb. Pore diffusion is the main transport mechanism by which mAbs bind to protein A resin.^45^ Unused MabSelect SuRe exhibited the highest binding capacity and a homogeneous binding profile regardless of mAb concentration loaded, indicating that all SPA binding sites are accessible within unused resin beads. In contrast, the used resins exhibit heterogeneous protein binding when loaded with mAbs at concentrations below saturation (Figure 3B). It is probable that as the resin has only been used for 25 cycles the level of contaminants makes some of the locations of the resin beads less accessible but does not block them entirely. Subsequent loading of subsaturating concentrations of mAb results in the individual mAb molecules preferentially binding to those SPA sites that are most easily accessible. This is supported by a study by Zhang et al.,^44^ who have shown that MabSelect SuRe resin beads exhibit decreased pore size and porosity after use. However, once a higher concentration mAb solution is applied, pore diffusion is able to overcome resistance caused by reduced pore size and therefore a homogeneous profile is observed in all resins once saturated with mAb (Figure 4A). This hypothesis is supported by the homogeneous diffusion model observed in polishing steps at saturation by Xiao et al.^36^ as well a study by Perez-Almondovar^45^ showing that large pore size reduces diffusional hindrance.

The Raman spectra, and in particular the presence of the Phe bands, indicated that irreversibly bound proteins that contain Phe are the main foulant in used protein A resins. Mass spectrometry analysis confirmed the presence of irreversibly bound mAbs as well as large amounts of peroxidases (categorized as HCP^43^) in the used resins. Peroxidases have been previously shown to interact more strongly with mAbs when compared to other HCPs and to coelute with mAbs during protein A purification.^43^ It was previously suggested that mAb based contaminants act as a nucleation point for further HCP accumulation and this increases with repeated resin use.^23^ Our findings further support this theory with a variety of HCPs being detectable in substantial abundance after just 25 cycles of purification. Further resin use up to 75 cycles shows no further mAb accumulation but a substantial further increase in HCP detection compared to the 25 cycle industrially fouled samples, suggesting that there is a defined process of contaminant build up with initial irreversible mAb binding leading to HCP accumulation.

The combination of Raman spectroscopy and mass spectrometry provides a unique insight into the identity of irreversibly bound contaminants and how the presence of these molecules on the resin does not just affect overall static binding capacity within a column but also binding of the mAb within individual resin beads. With our current setup it is not possible to use a macroscopic optical path that would allow measurement of a large number of beads simultaneously. While it is theoretically possible to perform such an analysis the increased amount of back scatter obtained from the spherical and nonuniform sized beads would introduce a markedly higher amount of background in resultant spectra.

Our findings suggest that new CIP procedures need to be designed to allow effective removal of the mAb foulant. Such procedures have the potential to extend the protein A resin lifetime and make mAb production more economical. Furthermore, our study shows that confocal Raman spectroscopy is a very powerful tool for monitoring and managing changes in resin binding capacity. The current study does indeed build on our earlier work but takes it a significant step further. The combination of Raman spectroscopy and mass spectrometry has provided a unique insight into the identity of irreversibly bound contaminants and how the presence of these molecules on the resin does not just affect overall static binding capacity within a column but also binding of the mAb within individual resin beads. These findings have the potential to inform new ways of working for large-scale therapeutic antibody production.

## Supporting Information Available

Photomicrographs of the resin beads (Figure S1), experimental setup (Figure S2), determination of surface of individual resin bead (Figure S3), confocal Raman intensity spectra of resin beads (Figure S4), PLS loading plots (Figure S5), actual vs predicted plot concentrations (Figure S6), frequency of peptide detected by MS (Figure S7), Raman spectra of resin samples (Figure S8), and HCPs detected in resin samples (Table S1) (PDF)
