# Supplementary material for: Causes of Industrial Protein A Column Degradation, Explored Using Raman Spectroscopy
Source: Anal Chem. 2022 Nov 1;94(45):15703–10. doi: 10.1021/acs.analchem.2c03063 (PMC9670029; doi:10.1021/acs.analchem.2c03063)
Supplement: Supplementary file 1 — ac2c03063_si_001.pdf [file ac2c03063_si_001.pdf]

Supplementary Information

Causes of industrial protein A column degradation, explored using Raman spectroscopy

James W. Beattie[1,2], Alena Istrate[3], Annabelle Lu[1], Cameron Marshall[1], Ruth C. Rowland-Jones[3], Monika Farys[3]†, Sergei G. Kazarian*[2], Bernadette Byrne*[1]

[1]Department of Life Sciences, Imperial College London, UK, SW7 2AZ; [2]Department of Chemical Engineering, Imperial College London, UK, SW7 2AZ,[3] Biopharm Process Research, Medicine Development & Supply, GSK R&D, Gunnels Wood Road, Stevenage, Hertfordshire, UK, SG1 2NY.

*Corresponding: b.byrne@imperial.ac.uk; s.kazarian@imperial.ac.uk

Table of contents

1. Figure 1 Light micrographs of the resin beads. S2
2. Figure 2 Schematic of experimental setup for mAb quantification used in this work. S3
3. Figure 3 Determination of the surface of an individual protein A resin bead using confocal Raman spectroscopy S4
4. Figure 4 Confocal Raman intensity spectra of unused MabSelect SuRe and used MabSelect SuRe from the inlet and outlet of a used 981 ml column. S5
5. Figure 5 PLS loading plots S6
6. Figure 6 Actual vs predicted plot concentrations of mAbs bound to protein A affinity resin samples. S7
7. Figure 7 LC-MS/MS analysis showing the frequency of detection of peptides in protein A resin after 75 cycles of purification with no mAbs loaded. S8
8. Figure 8 Raman spectra of resin samples S9
9. Table 1 HCPs detected by LC- MS/MS in inlet, outlet and 75 cycle resin samples. S10

Figure S1

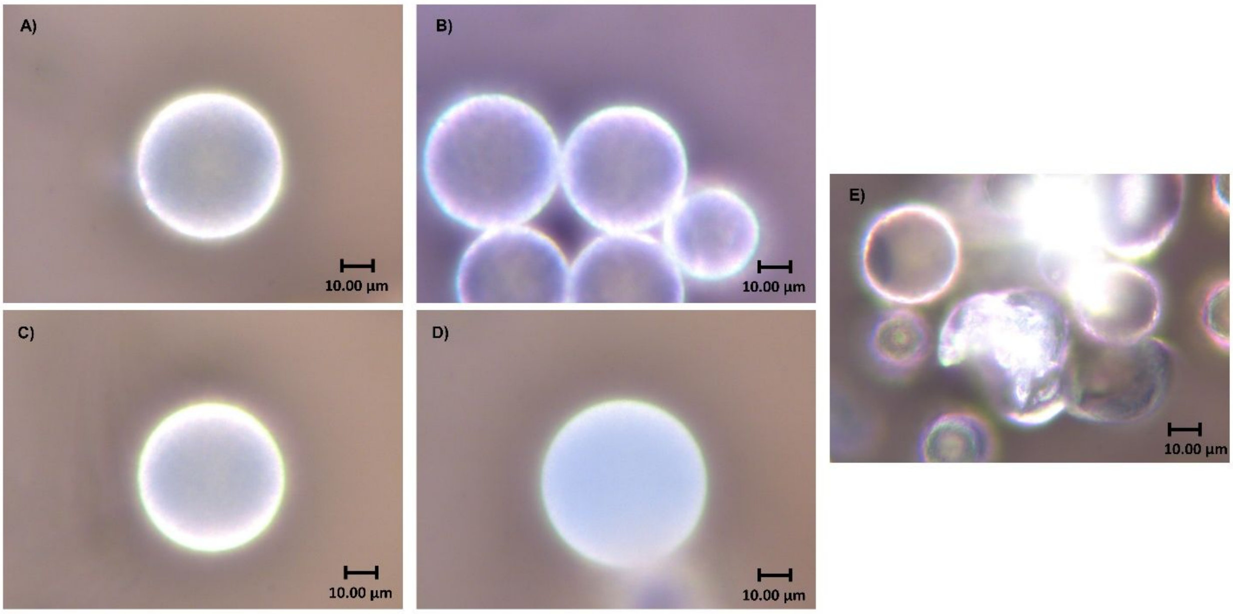

Light micrographs of the resin beads following transfer to glass slides for confocal Raman analysis using a polypropylene spreader. The images show the beads are intact after transfer using this approach and illustrate the variation in bead size. The manufacturers state bead size can be up to 120 µm in diameter. In our analysis most beads were found to be between 40 µm and 70 µm. A) MabSelect SuRe bead of 43.7 µm diameter B) MabSelect SuRe beads saturated with mAbs (65.3 mg ml^{-1}). The bead that was analysed was 40.4 µm in diameter. C) Used outlet bead of 46.9 µm diameter. D) Used inlet sample, the analysed bead had a diameter of 44.6 µm. E) Broken MabSelect SuRe beads obtained when transferring without the use of a polypropylene spreader.

Figure S2

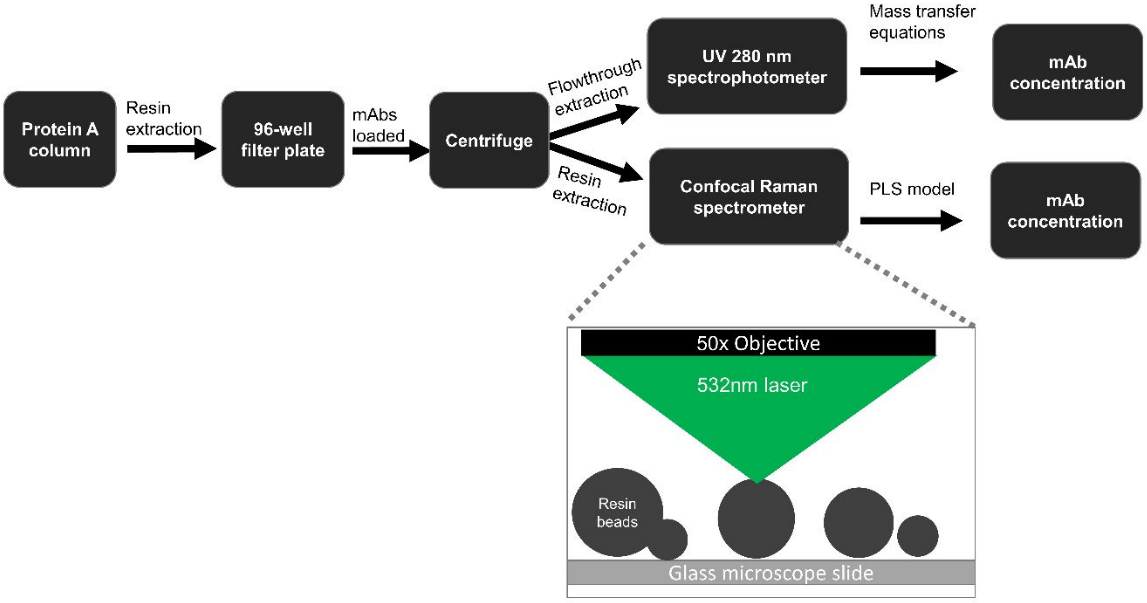

Schematic of experimental setup for mAb quantification used in this work.

Figure S3

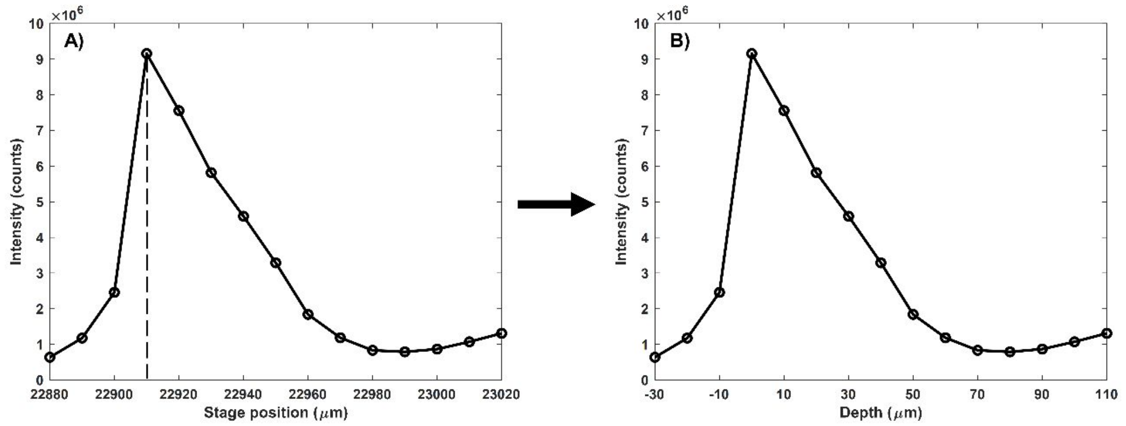

Determination of the surface of an individual protein A resin bead using confocal Raman spectroscopy. A) Intensity vs Stage position (distance lens position moved in z direction) plot obtained for a used outlet sample bead. The dotted line represents maximum spectral intensity and thus indicates the surface of the sample. B) Intensity vs depth plot for a used outlet resin bead once the stage position has been converted to depth in relation to the surface; 0 µm represents the surface of the resin bead. The resin bead was optically measured to be 53.45 µm in diameter. The Raman spectra were rubber band baseline corrected between 600 cm^{-1} and 1800 cm^{-1} followed by integration between 600 cm^{-1} and 1800 cm^{-1}. Maximal intensity was chosen in preference to FWHM. In A) above the half maximum is ~4.5*106 counts. The closest measured point to this was at stage position 22900 µm which is lower intensity than that obtained by focusing the beam past the sample (stage position 22950 µm). No clear spectral features can be observed at the depth closest to the half maximum (22900 µm) intensity. Most photons are coming from beyond the focal plane as the laser is still focused above the sample. We optically determined a point above the surface based on focus of the sample then probed in 10 µm steps into the sample.

Figure S4

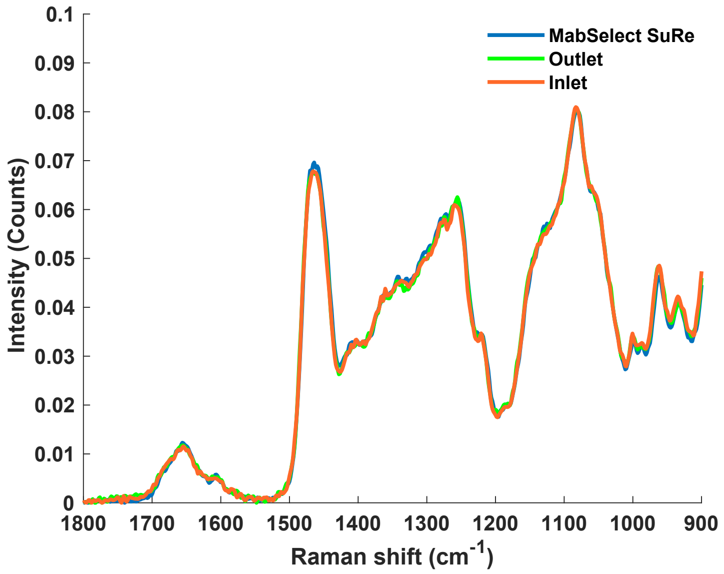

Confocal Raman intensity spectra of unused MabSelect SuRe and used MabSelect SuRe from the inlet and outlet of a used 981 ml column. The region 1800 cm^{-1}-900 cm^{-1} of the spectra are shown. There are no significant changes in the amide I bands between samples. Spectra is representative of N=2.

Figure S5

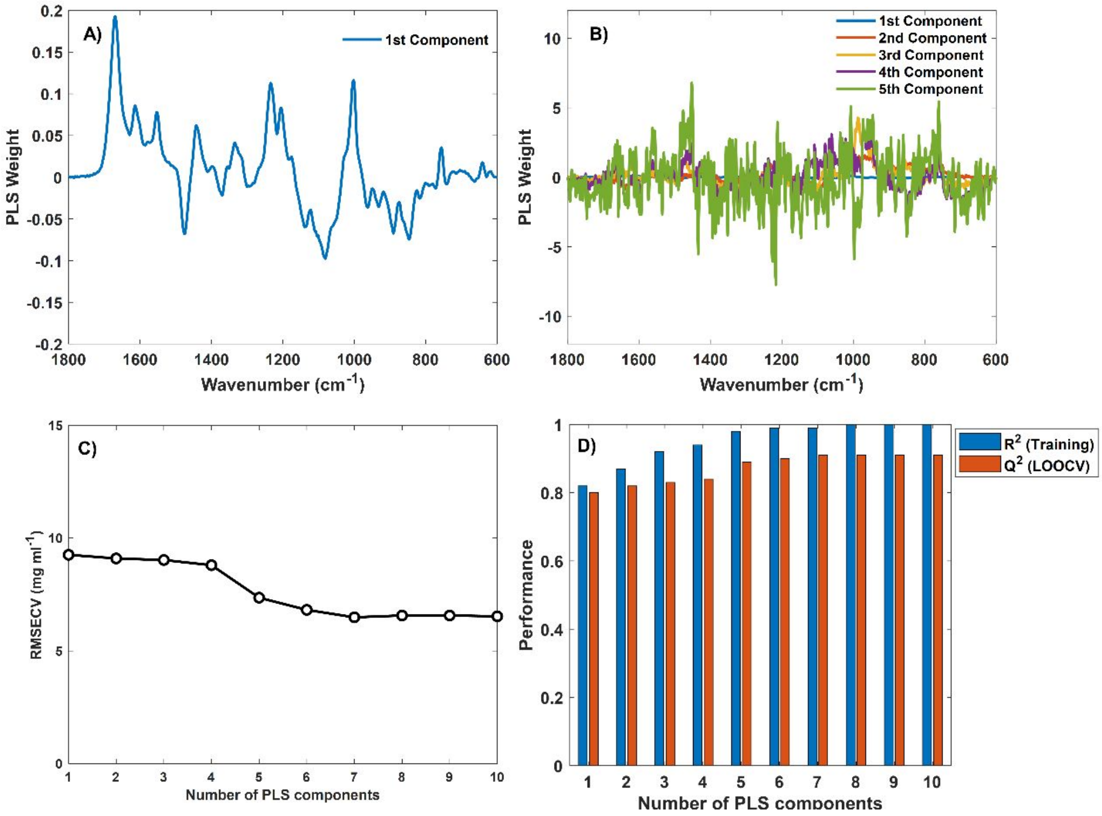

PLS loading plots A) PLS loading plot of the 1st component. B) PLS loading plot of components 1-5. C) Root mean squared error (RMSE) of the PLS model with increasing number of PLS components. The RMSE cross validation (RMSECV) was calculated by plotting the fitted vs observed model for each component using LOOCV (Leave one out cross validation). D) The R^2 Vs Q^2 plot. R^2 was calculated from the training data set and Q^2 was calculated from LOOCV. The RMSE plot in panel C shows a 7 component PLS model to be best for predicting mAb concentration. The Q^2 is maximum at 7 components in panel D, however the increase in Q^2 was very small for components 6 and 7 suggesting overfitting if more than 5 components were used. The loading plot becomes increasingly noisy as each component explains less and less variance in Y (mAb concentration) as indicated in panel B. Thus 5 components were chosen for the PLS model in order to prevent overfitting.

Figure S6

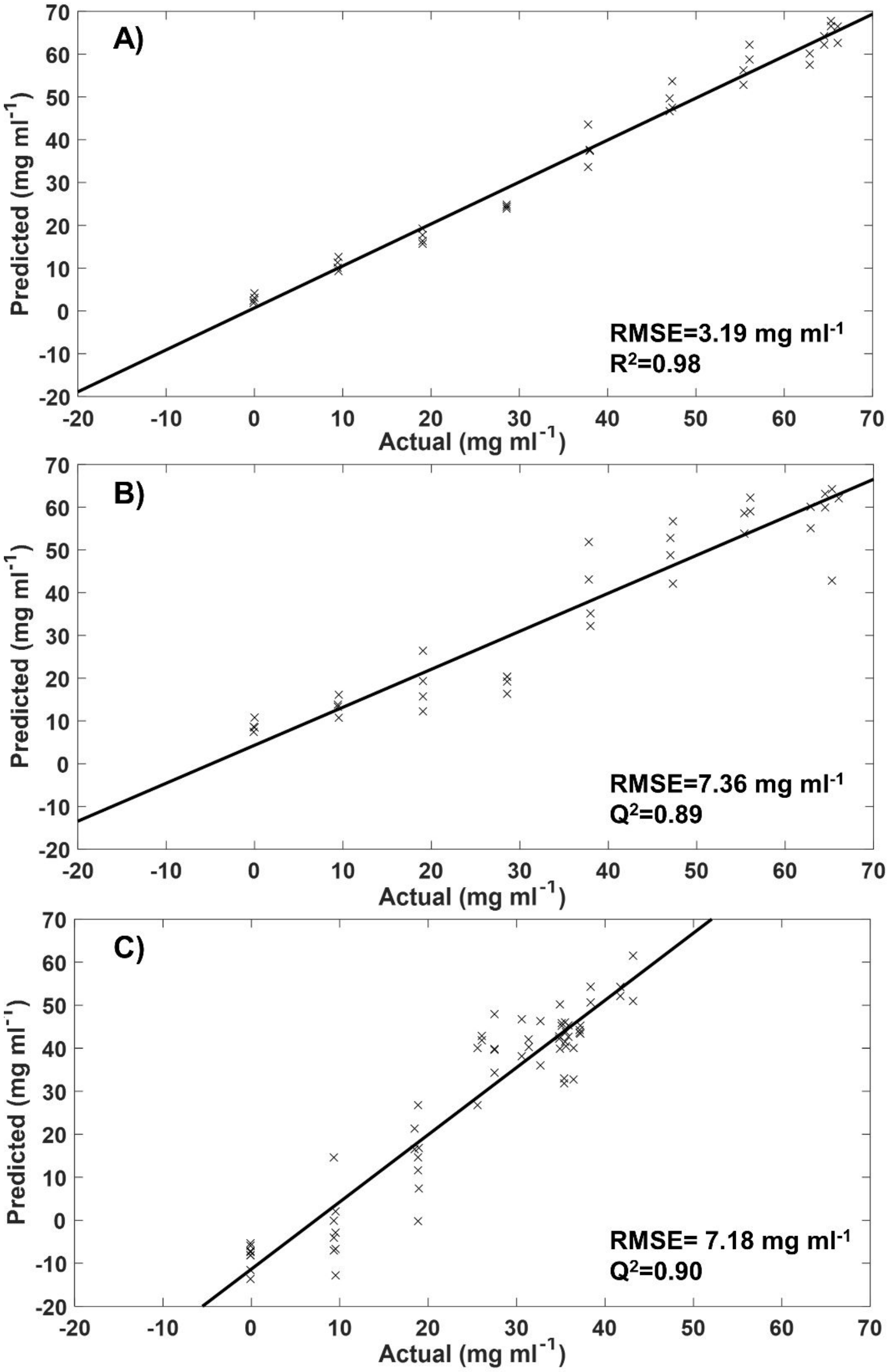

Actual vs predicted plot concentrations of mAbs bound to protein A affinity resin samples. A) training data, B) leave one out cross validation and C) test data. Actual is the Q value from SBC measurements and predicted was obtained from the Raman PLS regression model.

Figure S7

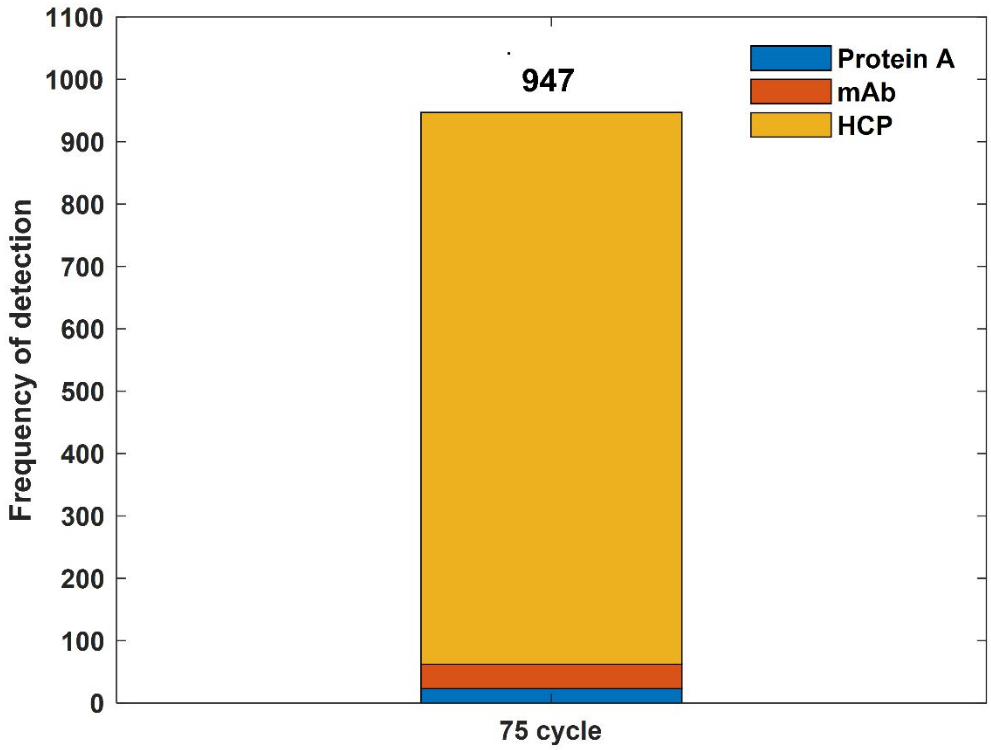

LC-MS/MS analysis showing the frequency of detection of peptides in protein A resin after 75 cycles of purification with no mAbs loaded. Peptides detected for each sample are grouped into category's Protein A (blue), mAb (red), HCP (yellow). HCP in this figure refers to all identified peptides that are not protein A or IgG based. A total of 947 peptides were detected with 23 being protein A 39 being IgG and 885 being HCP. There is a large increase in HCP when compared to inlet and outlet resin samples (25 cycles of purification). mAb and protein A showed one more peptide detection than the GSK inlet and outlet samples indicating protein A and mAb presence remained consistent regardless of cycle number.

Figure S8

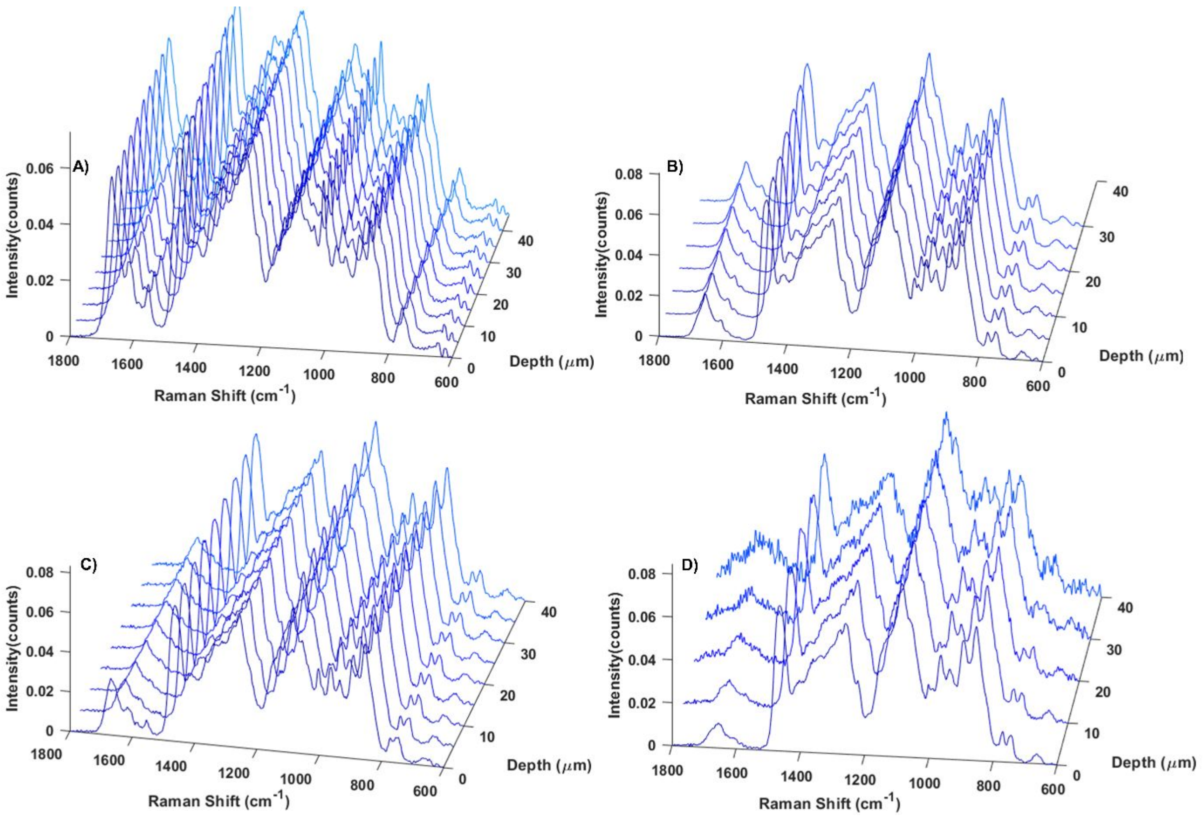

Raman spectra of resin samples. Plotted as a function of depth from the surface. A) MabSelect Sure saturated with mg ml^{-1} of mAb. B) MabSelect Sure saturated on average with 65.68 mg ml^{-1} of mAb. C) Outlet resin bead partially saturated with mean concentration of 18.85 mg ml^{-1} of mAb D) inlet resin bead with no mAbs loaded.

Table S1 HCPs Detected by LC- MS/MS in inlet, outlet and 75 cycle resin samples. X denotes presence of protein in sample.

	Inlet (25 cycles)	Outlet (25 cycles)	75 cycle sample
Endoplasmic reticulum chaperone BiP			X
Rab GDP dissociation inhibitor			X
Prolyl-tRNA synthetase (Fragment)			X
60S ribosomal protein L13			X
Importin-5			X
Sushi repeat-containing protein SRPX			X
Beta'-coat protein			X
T-complex protein 1 subunit theta			X
T-complex protein 1 subunit theta			X
Trifunctional purine biosynthetic protein adenosine-3			X
60S acidic ribosomal protein P0			X
Transketolase			X
ACTB	X	X	X
Ubiquitin	X		X
Arginine--tRNA ligase, cytoplasmic			X
Solute carrier family 2, facilitated glucose transporter member 9			X
Metavinculin			X
3-hydroxyacyl-[acyl-carrier-protein] dehydratase	X		X
Aspartate carbamoyltransferase			X
Cullin-associated NEDD8-dissociated protein 1			X
60S ribosomal protein L9			X
RNA helicase			X
Peroxiredoxin-1	X	X	X
40S ribosomal protein S8			X
Staphylococcal nuclease domain-containing protein			X
Phosphoglycerate mutase 1			X

Alpha-mannosidase			X
40S ribosomal protein S18			X
Coiled-coil domain-containing protein 80			X
Ribosomal protein L15			X
Alpha-actinin-1			X
Tubulointerstitial nephritis antigen-like	X	X	X
Histone H3			X
Septin-9			X
Pyruvate kinase	X	X	X
HSPA2	X		X
Importin subunit beta-1			X
Hexokinase			X
Alpha-1,4 glucan phosphorylase			X
Galectin			X
CCT-epsilon	X		X
Coatomer subunit alpha			X
Transforming protein RhoA			X
Phospholipid transfer protein	X		X
ATP-binding cassette sub-family E member 1			X
Cytoplasmic dynein 1 heavy chain 1 (Fragment)			X
Heat shock protein HSP 90-alpha			X
Peptidyl-prolyl cis-trans isomerase			X
Nucleoside diphosphate kinase			X
Peroxidase	X	X	X
Heat shock protein HSP 90-beta	X	X	X
Non-specific serine/threonine protein kinase	X		X
T-complex protein 1 subunit delta			X
Histone H2A		X	X
60S acidic ribosomal protein P0			X
Filamin-B			X
Tyrosine-protein kinase receptor	X		
T-complex protein 1 subunit gamma	X		X
Small nuclear ribonucleoprotein Sm D1			X

L-lactate dehydrogenase A chain			X
CCT-alpha	X		X
Laminin subunit alpha-5		X	
Elongation factor 1-alpha	X	X	X
Collagen alpha-1(XII) chain (Fragment)		X	
Elongation factor 1-alpha 1	X		X
Thrombospondin-1	X		X
Basement membrane-specific heparan sulfate proteoglycan core protein	X	X	X
Peptidyl-prolyl cis-trans isomerase			X
Clathrin heavy chain			X
Alpha-actinin-4 (Fragment)			X
Alpha-actinin-4			X
40S ribosomal protein S16			X
Spectrin beta chain, brain 1			X
Guanine nucleotide-binding protein subunit beta-2-like 1	X	X	X
40S ribosomal protein S3a			X
Junction plakoglobin	X		
ATP citrate synthase			X
Synaptic vesicle membrane protein VAT-1-like			X
Glyceraldehyde-3-phosphate dehydrogenase	X	X	X
60S ribosomal protein L7			X
60S ribosomal protein L11			X
GPI ethanolamine phosphate transferase 3			X
Clusterin	X	X	X
Phosphoglycerate kinase			X
Phosphoglycerate kinase			X
Isoleucyl-tRNA synthetase			X
D-3-phosphoglycerate dehydrogenase			X
GTP-binding nuclear protein Ran			X
Histone H4		X	X
Protein-synthesizing GTPase			X
Endoplasmin			X
Tubulin beta chain	X		X
40S ribosomal protein SA	X		X
Lipase	X		
Protein arginine N-methyltransferase 5			X

Matrix metalloproteinase-19			X
Elongation factor 2	X	X	X
Cytoplasmic FMR1-interacting protein			X
Lysosomal alpha-glucosidase	X		
F-actin-capping protein subunit beta			X
Adenylosuccinate lyase		X	X
Phosphoglycerate kinase	X		X
T-complex protein 1 subunit eta	X		X
CCT-beta	X		X
Tenascin-X		X	
Tenascin-X		X	
Ribosomal protein			X
40S ribosomal protein S4			X
60S ribosomal protein L13a			X
40S ribosomal protein S11			X
Elongation factor 1-gamma	X		X
Glyceraldehyde-3-phosphate dehydrogenase		X	X
Fibronectin			X
40S ribosomal protein S20			X
T-complex protein 1 subunit zeta			X
GST class-pi	X	X	X
Fructose-bisphosphate aldolase			X
60S ribosomal protein L7a			X
Tubulin alpha chain	X		X
Phospholipase B-like			X
Importin-7			X
Keratin, type I cytoskeletal 19	X		X
Keratin, type I cytoskeletal 14	X	X	X
Keratin, type I cytoskeletal 17	X		X
Keratin, type I cytoskeletal 42	X		
Triosephosphate isomerase			X
L-lactate dehydrogenase			X
26S proteasome non-ATPase regulatory subunit 2			X
40S ribosomal protein S9			X
Procollagen C-endopeptidase enhancer 1			X
AP-2 complex subunit alpha			X
2-phospho-D-glycerate hydro-lyase	X	X	X
Glutaminyl-tRNA synthetase			X

Filamin-A			X
Keratin, type II cytoskeletal 6A	X	X	X
Keratin, type II cytoskeletal 71	X	X	X
Aldose reductase-related protein 2	X		
Elongation factor 1-gamma			X
Ras GTPase-activating-like protein IQGAP1			X
Heat shock cognate 71 kDa protein			X
60S ribosomal protein L34			X
GTP-binding nuclear protein Ran			X
6-phosphogluconate dehydrogenase, decarboxylating	X		X
Keratin, type II cytoskeletal 5	X		
Glutathione transferase		X	X
40S ribosomal protein S15a			X
Pigment epithelium-derived factor			X
Glutathione S-transferase	X	X	
Uncharacterized protein			X
UDP-glucose 6-dehydrogenase	X	X	X
